# Pathogenesis and sexual transmission of Spondweni and Zika viruses

**DOI:** 10.1371/journal.pntd.0005990

**Published:** 2017-10-06

**Authors:** Erin M. McDonald, Nisha K. Duggal, Aaron C. Brault

**Affiliations:** Division of Vector-borne Diseases, National Center for Emerging Zoonotic Infectious Diseases, Centers for Disease Control and Prevention, Fort Collins, Colorado, United States of America; Weill Cornell Medical College, UNITED STATES

## Abstract

The Spondweni serogroup of viruses (*Flaviviridae*, *Flavivirus*) is comprised of Spondweni virus (SPONV) and Zika virus (ZIKV), which are mosquito-borne viruses capable of eliciting human disease. Numerous cases of ZIKV sexual transmission in humans have been documented following the emergence of the Asian genotype in the Americas. The African ZIKV genotype virus was previously implicated in the first reported case of ZIKV sexual transmission. Reports of SPONV infection in humans have been associated with non-specific febrile illness, but no association with sexual transmission has been reported. In order to assess the relative efficiency of sexual transmission of different ZIKV strains and the potential capacity of SPONV to be sexually transmitted, viral loads in the male reproductive tract and in seminal fluids were assessed in interferon α/β and –γ receptor deficient (AG129) mice. Male mice were inoculated subcutaneously with Asian genotype ZIKV strains PRVABC59 (Puerto Rico, 2015), FSS13025 (Cambodia, 2010), or P6-740 (Malaysia, 1966); African genotype ZIKV strain DakAr41524 (Senegal, 1984); or SPONV strain SAAr94 (South Africa, 1955). Infectious virus was detected in 60–72% of ejaculates collected from AG129 mice inoculated with ZIKV strains. In contrast, only 4% of ejaculates from SPONV-inoculated AG129 males were found to contain infectious virus, despite viral titers in the testes that were comparable to those of ZIKV-inoculated mice. Based on these results, future studies should be undertaken to assess the role of viral genetic determinants and host tropism that dictate the differential sexual transmission potential of ZIKV and SPONV.

## Author summary

The Spondweni serogroup of viruses, which includes Zika virus and Spondweni virus, are mosquito-borne viruses that can cause disease in humans. During the recent outbreak of Zika virus in the Americas, sexual transmission and *in utero* transmission have also been described. Due to the close genetic identity of Zika and Spondweni viruses, the herein reported study used a mouse model to assess the sexual transmission capacity of Spondweni virus in comparison to recent outbreak Zika strains and older Zika virus strains. In this model, all Zika strains were shed in seminal fluids from infected males. However, the percentage of ejaculates that contained infectious virus was significantly lower for Spondweni-infected males than Zika-infected males. Thus, sexual transmission potential is conserved among Zika viruses and not likely to fully explain the magnitude and dynamics of the recent outbreak in the Americas. In addition, sexual transmission potential should be further evaluated for Spondweni virus. Virus-specific differences in rates of shedding in seminal fluids will inform future studies on the viral determinants of sexual transmission.

## Introduction

Zika virus (ZIKV) and Spondweni virus (SPONV) are the only members of the Spondweni serogroup of mosquito-borne viruses (*Flaviviridae*, *Flavivirus*). Since the initial isolation of SPONV in 1952 [1], there have been at least five laboratory confirmed cases of SPONV infection in humans [2–5], although due to cross-reactivity in neutralization tests some reports of SPONV infection have been misdiagnosed as ZIKV infection [6]. Two genotypes of ZIKV (African and Asian) have been described and implicated with sexual transmission [7, 8]. Although both genotypes have been associated with human disease, the 2007 Yap island outbreak and the epidemic emergence of ZIKV in the Americas initiated in 2015 have been due to circulation of the Asian genotype ZIKV [9, 10]. Infectious ZIKV has been cultured from the semen of men infected during ZIKV outbreaks in French Polynesia [11] and the Americas for up to 24 days post-onset of disease [12], with viral RNA detection evident in semen for more than 6 months post-onset of disease [13, 14]. Some epidemiological studies have reported a higher incidence of ZIKV observed in women during the American and Asian outbreaks [15, 16], suggesting that male-to-female sexual transmission could account for this gender bias.

Animal models of ZIKV sexual transmission have been developed in immunodeficient mice, with evidence of sexual transmission from male mice inoculated with an Asian genotype of ZIKV to female mice [17–19]. Infectious virus has also been detected in seminal fluids collected from ZIKV-inoculated interferon α/β and –γ receptor knockout (AG129) mice between 7 and 21 days post-inoculation. Detectable infectious virus during this time period was present at the same frequency (51%) as sexual transmission events from infected male mice to females was observed (50%), thus establishing this as a model to measure sexual transmission potential [17]. In addition, ZIKV tropism for the male reproductive tract has been described in several immunodeficient mouse models, including A129 mice, AG129 mice, C57BL/6 *Ifnar1-/-* mice, and C57BL/6 *Rag1-/-* mice treated with a monoclonal antibody to Ifnar1 [19–22]. The relationship between infection of the testes/epididymides and sexual transmission potential has not been fully established, though vasectomized male AG129 mice have demonstrated a reduced magnitude of virus in seminal fluid compared to non-vasectomized males[17]. Tissue tropism and pathogenesis of SPONV in mice is unknown, though it is known to cause death in newborn and weanling immune competent mice after intracranial inoculation [23].

To assess the sexual transmission potential of SPONV and the African and Asian genotypes of ZIKV, seminal fluids were collected from inoculated male AG129 mice as described previously [17]. Herein, the African and Asian genotype ZIKV strains collected from 1966 to 2015 showed a similar tissue tropism and sexual transmission potential in AG129 mice, suggesting sexual transmissibility is not a recently acquired transmission phenotype of ZIKV. However, SPONV had a significantly lower potential for sexual transmission, with only 4% of seminal fluids containing infectious virus, despite SPONV having a similar tissue tropism and titers in the male reproductive tract as ZIKV in AG129 mice.

## Results

### Pathogenesis and tissue tropism of SPONV and Asian and African ZIKV strains in immunodeficient mice

**Fig 1:**
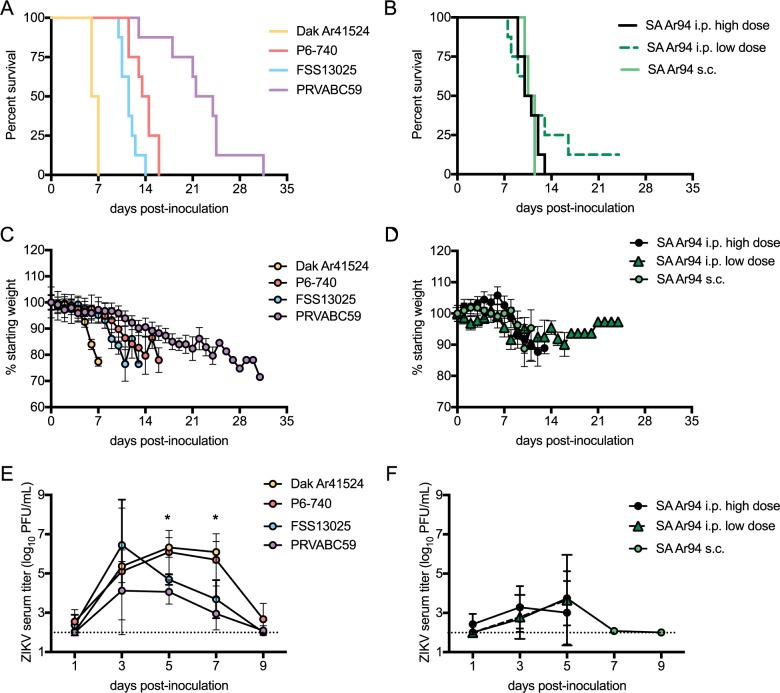
Pathogenesis and viremia of ZIKV and SPONV in male AG129 mice. Mice were inoculated s.c. with 3 log_10_ PFU of ZIKV strains PRVABC59, P6-740, FSS13025, DakAr41524 (n = 8 per virus strain), or SPONV strain SA Ar94 (n = 4), or inoculated i.p. with SPONV strain SA Ar94 at two doses, either 5.4 log_10_ PFU or 3 log_10_ PFU (n = 8 each). (A) Survival curve for mice inoculated with the ZIKV strains. DakAr41524 vs. any other ZIKV strain (p<0.05); PRVABC59 vs. any other ZIKV strain (p<0.05). (B) Survival curve for mice inoculated with the SPONV strain. SA Ar94 vs. DakAr41524 and PRVABC59 (p<0.001). (C) Weight of mice inoculated with the ZIKV strains, shown as a percentage of initial weight. (D) Weight of mice inoculated with the SPONV strain, shown as a percentage of initial weight. (E) Mean viremia of mice inoculated with ZIKV strains. Dpi 5 and 7: P6-740 and DakAr41524 vs. PRVABC59 and FSS13025 (*, p<0.05). (F) Mean viremia of mice inoculated with the SPONV strain. Dpi 3: SA Ar94 vs. any ZIKV strain (p<0.05); dpi 5 and 7: SA Ar94 vs. DakAr41524 and P6-740 (p<0.01). Error bars represent standard deviations from the mean.

The pathogenesis and tissue tropism of four different ZIKV strains representing both genotypes and one SPONV strain were assessed in the AG129 mouse model (Fig 1). Three low-passage strains representing the Asian genotype of ZIKV (PRVABC59, Puerto Rico 2015; P6-740, Malaysia 1966; and FSS13025, Cambodia 2010) and a low-passage strain representing the African genotype of ZIKV (DakAr41524, Senegal 1984) were inoculated subcutaneously (s.c.) into the footpad of AG129 male mice. For SPONV, two different infection routes were compared in AG129 mice: SA Ar94 (South Africa, 1955) was inoculated intraperitoneally (i.p.) at two different doses, or inoculated s.c. Mice were observed daily, and their weights recorded until the mice met euthanasia criteria (paralysis, loss of 20% of body weight, or reduced mobility).

The African genotype virus, DakAr41524 strain, was the most pathogenic ZIKV strain in AG129 mice, with inoculated mice exhibiting a median survival time of 6.5 days. The Asian genotype ZIKV strains were less pathogenic, with median survival times of 11.5 days for FSS13025, 14 days for P6-740 and 22.8 days for PRVABC59. All survival curves for ZIKV, except P6-740 vs. FSS13025 in AG129 mice, were statistically significantly different using a family-wise significance level of 5% (Fig 1A). For SPONV-inoculated AG129 mice, the median survival time was not significantly different between the i.p. and s.c. groups, and the three groups were combined into a single group for analyses (Fig 1B). The mean survival time for SPONV-inoculated mice was 10.8 days, which was significantly different than the DakAr41524 and PRVABC59-inoculated mice (p<0.001), but not the P6-740- or FSS1325-inoculated mice. Mean weight loss greater than or equal to 5% of initial body weight was observed 2 to 13 days prior to the mice being euthanized (Fig 1C and 1D).

The viremia profiles of ZIKV strains in AG129 mice were similar between the two most recently isolated Asian genotype viruses (PRVABC59 and FSS13025) and peaked on day post-inoculation (dpi) 3 with mean titers of 4.1 and 6.4 log_10_ PFU/mL, respectively (Fig 1E). The viremia profiles in AG129 mice were similar between the older ZIKV strains (P6-740 and DakAr41524) and peaked on dpi 5 with mean titers of 6.1 and 6.3 log_10_ PFU/mL, respectively (Fig 1E). The mean viremias of P6-740- and DakAr41524-inoculated AG129 mice were higher than PRVABC59- and FSS13025-inoculated AG129 mice at dpi 5 and 7 (p<0.05). For mice inoculated by the i.p. route with the low and high dose of SPONV or inoculated s.c. with SPONV, mean peak serum viremias were statistically indistinguishable (3.3, 3.7, and 3.7 log_10_ PFU/mL, respectively) and were thus combined into a single group for analyses (Fig 1F). SPONV-inoculated mice had lower viremias than mice inoculated with any ZIKV strain at dpi 3 (p<0.05) and lower viremias on dpi 5 and 7 than the mice inoculated with the older ZIKV strains (p<0.01).

**Fig 2:**
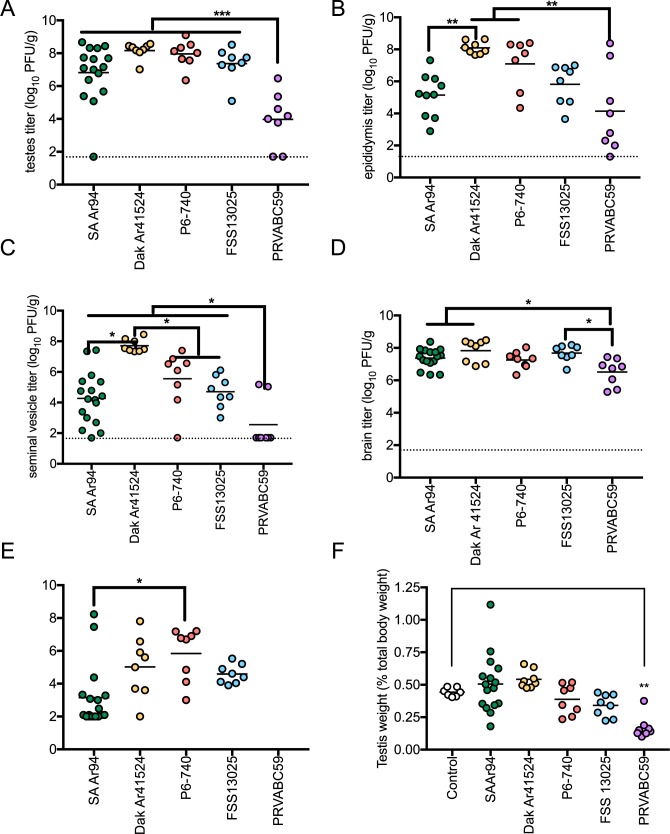
Tissue tropism of ZIKV and SPONV in male AG129 mice. Mice were inoculated s.c. with ZIKV (n = 8 per virus strain) or inoculated i.p. with a SPONV strain at two doses (n = 8 each) or s.c. (n = 4). The i.p. and s.c. groups for SPONV were combined into a single group for analyses. (A) Viral titers in the testes. PRVABC59 vs. any other strain (p<0.001). (B) Viral titers in the epididymis. PRVABC59 vs. DakAr41524 and P6-740 (p<0.01); SA Ar94 vs. DakAr41524 (p<0.01). (C) Viral titers in the seminal vesicles. PRVABC59 vs. any other ZIKV strain (p<0.05); DakAr41524 vs. any other strain (p<0.05). (D) Viral titers in the brain. PRVABC59 vs. FSS13025, DakAr41524, and SA Ar94 (p<0.05) (E) Viral titers in the eye. SA Ar94 vs. P6-740 (p<0.05). (F) Testicular weight as a percentage of total body weight. Control vs. PRVABC59 (p<0.01).

Tissue distribution of the ZIKV and SPONV strains was assessed by measuring infectious ZIKV or SPONV titers in serum, brain, testes, epididymides, seminal vesicles and eyes at the time of euthanasia. Since weight loss (Fig 1D), median survival time (Fig 1B) and viremia profiles (Fig 1F) were indistinguishable for the two doses of SPONV i.p.-inoculated mice and s.c.-inoculated mice, SPONV titers in tissues collected at time of euthanasia were combined for these groups. Overall, PRVABC59-inoculated mice exhibited lower mean viral titers at the time of euthanasia in organs of the male reproductive tract and brain tissue than other ZIKV and SPONV strains (Fig 2A–2D, p<0.05), which is likely explained by the longer survival time of these mice. SPONV strain SA Ar94 shared a similar tissue tropism as ZIKV strains in AG129 mice for the male reproductive tract, with statistically indistinguishable titers in the testes, epididymis, and seminal vesicles compared to P6-740 and FSS13025, which are the ZIKV strains with the most similar survival times to SPONV. The mean eye titer did not differ among ZIKV strains, although SPONV-inoculated mice had significantly lower mean titers in the eyes than that observed in P6-740-inoculated mice (p<0.05).

Because previous studies have described testicular atrophy in mice inoculated with ZIKV [18, 24], testis weight was compared between ZIKV-inoculated mice and SPONV-inoculated mice. A testis from each mouse was weighed at time of euthanasia, and testis weight as a percentage of the mouse’s starting weight was compared to non-inoculated control mice. The weight of testes from mice inoculated with PRVABC59 were significantly lower compared to those of control mice (Fig 2F, p<0.01). The testes of mice inoculated with either SPONV, the other Asian ZIKV strains, or the African strain of ZIKV were not significantly different in weight than control mice, but this is likely due to the rapid mortality of these mice.

### Sexual transmission potential of SPONV and Asian and African ZIKV strains

Previous work demonstrated that infectious PRVABC59 ZIKV could be found in the ejaculates of male AG129 mice inoculated by the i.p. route from dpi 7 to 21 [17]. To compare the viral kinetics of African and Asian genotype ZIKV strains and SPONV in seminal fluids, ejaculates were collected beginning at 5 dpi from male AG129 mice inoculated with the ZIKV strains or SPONV strain. Ejaculates were collected from male mice by mating them to female immune competent CD-1 mice and flushing the contents from the uteri of mated female mice as described previously [17].

**Fig 3:**
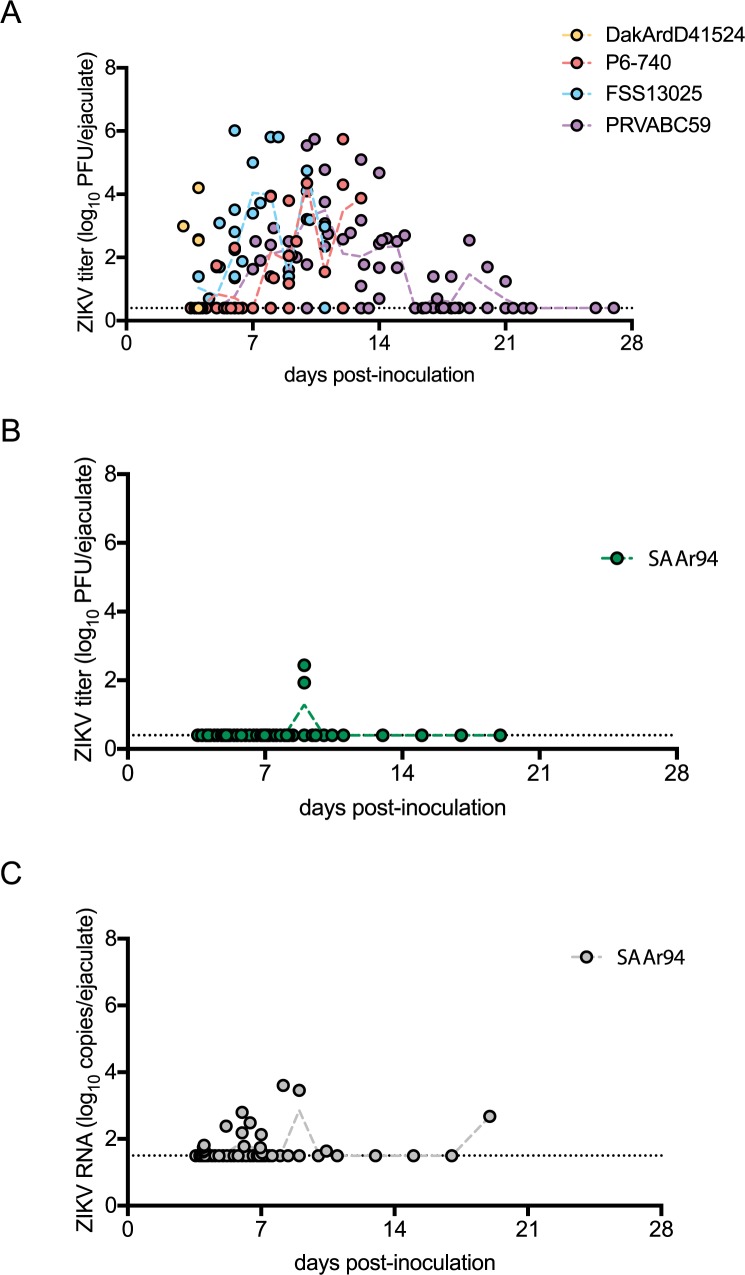
Sexual transmission potential of ZIKV and SPONV in male AG129 mice. (A) Viral titers in seminal fluids collected from mice inoculated with ZIKV strains. (B) Viral titers in seminal fluids collected from mice inoculated with SPONV strain SA Ar94. (C) Viral RNA copy in seminal fluids collected from mice inoculated with SPONV strain SA Ar94.

The ZIKV-inoculated AG129 mice shed infectious ZIKV in seminal fluids beginning at dpi 5 or 6 (Fig 3A). Due to the rapid mortality of DakAr41524-inoculated mice, ejaculates were only collected on dpi 5. For FSS13025- or P6-740-inoculated mice, ejaculates were only collected through dpi 12 or 14 due to mortality. Ejaculates were collected from PRVABC59-inoculated mice through dpi 28, but ejaculates contained infectious virus only through dpi 23. During the period of infectivity, the percentage of ejaculates found to be positive for infectious virus was statistically indistinguishable for all ZIKV strains (70, 72, 63, and 60% for PRVABC59, FSS13025, P6-740, and DakAr41524, respectively; Table 1). The mean titer for ejaculates with infectious virus was not significantly different between ZIKV strains (3.2–5.0 log_10_ PFU/ejaculate; Table 1) and peaked on dpi 10–13. The timing of peak titers for DakAr41524-inoculated mice was not possible to ascertain due to rapid mortality.

**Table 1:** Summary of infectious range and titer of ejaculates.

Virus strain	# ejaculates collected (dpi range)	% ejaculates positive for infectious virus	Infectious range (dpi)	% ejaculates positive for infectious virus during infectious range (N)	Mean titer of infectious samples, log_10_ PFU/ejaculate (N)	Day of peak mean titer (dpi)
SA Ar94	50 (5–20)	4%	10	50% (4)	2.2 (2)	10
Dak Ar41524	5 (5)	60%	5*	60% (5)	3.2 (3)	5*
P6-740	27 (5–14)	52%	6–14	63% (24)	5.0 (15)	13
FSS13025	32 (5–12)	72%	5–12	72% (32)	4.7 (23)	11
PRVABC59	75 (5–28)	67%	5–23	70% (73)	3.7 (51)	11

*only time point assessed

dpi = days post inoculation

Only two of 50 (4%) ejaculates collected from the SPONV-inoculated mice were found to contain infectious virus (Fig 3B), which was significantly lower than the fraction of ejaculates collected from ZIKV-inoculated males (p<0.001). The two ejaculates with infectious virus were both collected on dpi 10 from two different males with a mean titer of 2.2 log_10_ PFU/ejaculate and represented 50% of ejaculates collected on dpi 10 (Table 1). The two males with ejaculates containing infectious virus were inoculated i.p. (high dose) and s.c. To confirm these results, ejaculates were tested by qRT-PCR for SPONV RNA. 37% of the SPONV ejaculates over the entire time course contained SPONV RNA, but only samples with at least 3.5 log_10_ RNA copies/ejaculate were found to contain infectious virus (Fig 3C). The two samples with infectious virus had an average RNA: PFU ratio of 1.6, which was very similar to previous estimates of 1.5 for the RNA: PFU ratio for ZIKV in mouse ejaculates during acute infection [17]. Thus, SPONV has sexual transmission potential, but with a lower efficiency due to low viral titers in seminal fluids.

## Discussion

The sexual transmission capacity of ZIKV is unique among known arboviruses that are transmitted to humans. Here we show that the most closely related virus, SPONV, is also capable of sexual transmission in a mouse model, but at a significantly lower rate. Infectious virus was detected in at least 60% of ejaculates collected from AG129 mice inoculated with either African and Asian genotype strains of ZIKV and was detected through dpi 23 (Fig 3). In contrast, only 4% of ejaculates from SPONV-inoculated AG129 mice contained infectious virus, and infectious virus was detected only on dpi 10 (Fig 3). The dissemination of SPONV in male mice was similar to ZIKV, as high viral titers were found in the male reproductive tract, including the testes, epididymides, and seminal vesicles (Fig 2), though transient viremia in SPONV-inoculated mice reached lower titers than in ZIKV-inoculated mice (Fig 1). Additionally, decreased testicular weight and persistent viral RNA in seminal fluids, which are characteristic of ZIKV infection in mice, were seen in SPONV-inoculated mice (Figs 2 and 3).

The African genotype ZIKV strain was significantly more pathogenic in AG129 mice than the Asian genotype ZIKV strains (Fig 1). The less pathogenic phenotype observed with the Asian genotype strains of ZIKV relative to the African genotype strain has been reported in other immunodeficient mouse models of ZIKV [25, 26] and indicates that the most recent ZIKV outbreak in the Americas was not likely due to a recent increase in ZIKV virulence. However, this does not preclude the potential that the Asian genotype ZIKV strains could be associated with other virulent disease processes such as congenital ZIKV syndrome by altered pathologic potential for neural progenitor cells [27]. Furthermore, the sexual transmission rates of the Asian and African genotype ZIKV strains were not significantly different, which indicates that sexual transmission is not a recently adapted transmission mechanism and is unlikely to have driven the American outbreak. This is supported by the first case of suspected sexual transmission, which was identified after a traveler was infected in Africa and transmitted to his partner upon returning to America [7]. In fact, sexual transmission of the Spondweni serogroup viruses may be a conserved transmission mechanism that could allow for short-term maintenance of the virus in the absence of competent vectors and could be a dissemination mechanism to increase the geographic range of these viruses. However, this is not necessarily a conserved mechanism across flaviviruses. Dengue virus serotypes 2, 3, and 4 have similar tropism to ZIKV and SPONV in AG129 mice, but tropism to the male reproductive tract has not been studied [28–30]. Immunocompetent mice or mice with transient immunodeficiency inoculated with DENV-2 were not found to have infectious virus in the testes or epididymides, and after intra-testicular injection of DENV-2 into Type I IFN receptor knockout mice, the damage to testicular architecture was reversed and spermatogenesis was observed [24, 31]. Thus, potential sexual transmissibility could be a restricted phenomenon to the Spondweni flavivirus serogoup.

A limitation of this study was the use of immunodeficient mice. Immunodeficient mouse models demonstrate more severe pathological outcomes, such as complete loss of spermatogenesis [31] following ZIKV infection, compared to immunocompetent mouse models. However, inoculation of ZIKV into immunocompetent mouse models following transient knockdown of Type I IFN-α/β receptor signaling, or inoculation of Type I IFN-α/β receptor knockout mice, results in infection of the male reproductive tract, and testicular atrophy[24, 31], thus supporting our observations in AG129 mice.

Human cases of sexually transmitted SPONV have yet to be described; however, as sexual transmission of ZIKV likely remained undetected for many years, future studies on the epidemiology of SPONV may identify cases of sexual transmission. While we have not assessed the susceptibility of female mice to intravaginal exposure of SPONV, and we only assessed one of two documented SPONV isolates, SPONV appears to be capable of sexual transmission in this mouse model, albeit for a much more limited time interval than ZIKV. The high testicular viral titers in SPONV-inoculated mice suggests a similar viral pathology to ZIKV-inoculated mice. However, shedding of infectious SPONV in seminal fluids occurred in AG129 mice at only one time point, which underscores a dissimilar underlying mechanism for sexual transmission potential between these viruses in this mouse model. A potential mechanism to explain these observed differences could be dissimilar tropism for host cells within the male reproductive tract, determined by viral genetic determinants, that results in differential sexual transmission efficiencies of the viruses. Future studies to assess the potential effects on male fertility between ZIKV and SPONV, delineate host cell populations required for sexual transmission, and to assess SPONV sexual transmission potential in non-human primates may provide additional insight into the mechanism(s) and host range of viral shedding in seminal fluids of viruses within the Spondweni serocomplex.

## Materials and methods

### Viruses

The virus isolates used in this study were: PRVABC59 (Puerto Rico 2015; Vero passage 3), P6-740 (Malaysia 1966; suckling mouse passage 6, Vero passage 3), FSS13025 (Cambodia 2010; Vero passage 4), DakAr41524 (Senegal 1984, AP61 passage 1, C6/36 passage 1, Vero passage 4), and SPONV strain SAAr94 (South Africa 1955, unknown host passage 6, Vero passage 2).Viruses were propagated and handled in BSL2 and ABSL2 laboratory conditions according to CDC guidelines [32].

### Inoculation of AG129 mice

Mice deficient in interferon α/β and -γ receptors (AG129 mice) were bred in-house, and the receptor knockout genotype of the mice was confirmed as described in [17]. 18-to 20-week-old male mice were inoculated s.c. with 10^3^ PFU of ZIKV strain PRVABC59, P6-740, FSS13025, DakAr41524, or 10^3^ PFU of SPONV strain SA Ar94. 16- to 18-week-old male mice were inoculated i.p. with either 5.4 log_10_ PFU (high dose) or 3 log_10_ PFU (low dose) of SPONV strain SA Ar94. Mice were euthanized when clinical evidence of disease was observed. Mice were euthanized after isoflurane-induced deep anesthesia followed by cervical dislocation. Tissues and serum were collected at time of euthanasia. For plaque assays, brain, eye, testes, epididymides, and seminal vesicles were collected, weighed and homogenized using a pestle in an equal volume of BA-1 medium, and then clarified by centrifugation and serially diluted for cell plaque assay to enumerate plaque forming units (PFU). For ZIKV-inoculated tissues and serum, the overlay for the Vero cell plaque assay was added four days post-inoculation. For SPONV-inoculated tissues and serum, the overlay for the LLC-MK2 cell plaque assay was added five days post-inoculation.

### Collection of seminal fluids from male AG129 mice

Seminal fluids from male AG129 mice were collected as described in [17]. In brief, inoculated male mice were housed individually, and each evening (beginning on dpi 5) five female CD-1 mice were introduced into the cage. The following morning, mating activity was assessed by determining whether a copulatory plug was identified in the female. If a copulatory plug was identified, the female was euthanized by isoflurane anesthetization followed by cervical dislocation. Both horns of the uterus were flushed with 500 uL of BA-1 media. Infectious ZIKV in the seminal fluids was titrated by Vero cell plaque assay, and infectious SPONV in the seminal fluids was titrated by LLC-MK2 plaque assay.

### SPONV RNA quantification

RNA was extracted from seminal fluid using the MagMax Viral RNA Isolation kit (Ambion), as described previously [17], with the exception that seminal fluids were not denatured in 10 mM DTT. A standard curve was generated by *in vitro* transcription of a plasmid containing a fragment of the SPONV strain SA Ar94 genome spanning nucleotides 3,291 to 4,357. The probe and primer sequence are as follows: Probe [6FAM]CATAGGACTGCTGGTGGTGA[TAM]; Forward primer (5’ AACCAAGACCGACATTGACA 3’); Reverse primer (5’ CACTCTTGCCAGAAACCACA 3’). All real-time assays were performed by using the QuantiTect Probe RT-PCR Kit (Qiagen, Valencia, CA, USA) with amplification in the Bio-Rad CFX96 Touch real-time PCR (Bio-Rad, Hercules, CA, USA) following the manufacturer’s protocol. The detection limit for this assay was 3 log_10_ RNA copies/mL (or 1.5 log_10_ RNA copies/ejaculate).

### Statistics

Survival curves were compared using a log-rank (Mantel-Cox) test. Viral titers in tissues and ejaculates and testicular weights were compared using ANOVA, and viral titers in serum were compared using multiple t-tests with a Holm-Sidak correction for multiple comparisons. Proportions of ejaculates containing infectious virus were compared using Fisher’s Exact Test. Statistical tests were performed in GraphPad Prism.

### Animal ethics statement

All experiments involving mice were approved by institutional animal care and use committee (IACUC) at the Division of Vector-Borne Diseases, Centers for Disease Control and Prevention under protocol 16–013. All protocols and practices for the handling and manipulation of mice were in accordance with the guidelines of the American Veterinary Medical Association (AVMA) for humane treatment of laboratory animals.
